# Long-term follow-up after bypass surgery or coronary stenting in elderly with multivessel disease

**DOI:** 10.1007/s12471-020-01415-z

**Published:** 2020-04-24

**Authors:** M. E. Gimbel, L. M. Willemsen, M. C. Daggelders, J. C. Kelder, T. Oirbans, K. F. Beukema, E. J. Daeter, J. M. ten Berg

**Affiliations:** Department of Cardiology and Cardiothoracic Surgery, St. Antonius Hospital, Nieuwegein, The Netherlands

**Keywords:** Revascularisation, Coronary artery disease, CABG, PCI

## Abstract

**Background:**

We sought to compare long-term follow-up of coronary artery bypass grafting (CABG) with percutaneous coronary intervention (PCI) in elderly patients with left main or multivessel disease, hypothesising that completeness of revascularisation and severity of coronary artery disease are predictors of adverse outcomes.

**Methods:**

Patients aged ≥75 years with multivessel disease or left main disease who underwent PCI or CABG between 2012–2016 were included in this retrospective cohort study. Baseline characteristics from the index procedure were collected. Severity of coronary artery disease and completeness of revascularisation were assessed. Primary outcome was all-cause mortality, in addition we captured major adverse cardiac and cerebral events, bleedings, recurrent angina and new onset atrial fibrillation.

**Results:**

A total of 597 patients were included. Median follow-up was 4 years (interquartile range 2.8–5.3 years). At baseline, patients in the PCI group more often had a previous medical history of CABG and more frequently underwent an urgent procedure compared with patients in the CABG group. Mortality at 5‑year follow-up was significantly higher in patients who underwent PCI compared with CABG (39.9% vs 25.4%, *p* < 0.001). Furthermore, acute coronary syndrome (ACS), repeat revascularisation and recurrent angina occurred more frequently after PCI, while occurrence of bleedings and new onset atrial fibrillation were more frequent after CABG. Neither completeness of revascularisation nor severity of coronary artery disease was a predictor for any of the outcomes.

**Conclusion:**

Long-term mortality was higher in elderly patients with multivessel disease undergoing PCI compared with CABG. In addition, patients undergoing PCI had a higher risk of ACS, repeat revascularisation and recurrent angina.

## What’s new

- Patients of 75 years or older with multivessel or left main disease have a significantly higher 5‑year mortality when treated by percutaneous coronary intervention (PCI) compared with coronary artery bypass grafting (CABG) with an adjusted hazard ratio of 1.59.
- Recurrent acute coronary syndrome, repeat revascularisation and recurrent angina all occurred significantly more frequently in these elderly patients who underwent PCI compared with CABG, while new onset atrial fibrillation and bleeding occurred significantly more often after CABG than after PCI.
- Completeness of revascularisation appeared not to be an independent predictor of adverse outcomes in this patient population.

## Background

Coronary artery bypass grafting (CABG) has long been the standard of care for patients with left main or multivessel disease. However, results of percutaneous coronary intervention (PCI) have been improved by better stents and more potent P2Y_12_-inhibitors. Therefore, the European Society of Cardiology (ESC) guideline for management of myocardial revascularisation now recommends either CABG or PCI based on individual decision making by the local heart team, taking into consideration operation risk, complexity of underlying coronary artery disease, intracardiac and extracardiac factors and local expertise. Furthermore, it is emphasised that achieving complete revascularisation is pivotal [1]. The ESC guideline does not advise on which revascularisation strategy is preferred in elderly patients in comparison with younger patients, probably because the optimal revascularisation treatment in elderly is unknown. PCI is less invasive with shorter hospital stay and earlier return to daily activities compared with CABG. This is particularly relevant for the elderly, in whom physical recovery after CABG procedures is substantially prolonged compared with younger patients [2]. Several observational studies have been conducted comparing PCI and CABG in the elderly (≥75 years) with multivessel disease and/or left main disease [3–6]. These studies found CABG to be associated with a significantly lower risk for target vessel revascularisation but found no significant difference in all-cause death. These studies were performed in patients treated with first generation drug-eluting stents and dual antiplatelet therapy consisting of aspirin with clopidogrel. Also, patients treated with PCI or CABG were not similar with respect to completeness of revascularisation and complexity of coronary artery disease. Therefore, aim of this study is to compare CABG with PCI in elderly (≥75 years) patients with multivessel or left main disease, considering completeness of revascularisation and severity of coronary artery disease.

## Methods

### Study design

We conducted a retrospective, single-centre cohort study in the St. Antonius hospital, the Netherlands. All patients aged ≥75 years with multivessel disease or left main disease who underwent revascularisation between January 1st, 2012 and December 31st, 2016 were included. Patients underwent revascularisation either by PCI or CABG, which was decided by a multidisciplinary heart team consisting of an interventional cardiologist and a cardiac surgeon. Patients who presented with ST-segment elevation myocardial infarction or who underwent emergency revascularisation were excluded. All patients were treated according to the applicable guidelines at that moment. The surgical technique for CABG, the approaches used for stent implantation, and medication regimen post revascularisation were left to the discretion of the treating physician. Patients were included if they had at least one year follow-up after the index procedure. Patients with follow-up in other hospitals were sent a questionnaire inquiring about recurrent revascularisation, myocardial infarction, cerebral vascular accident (CVA), bleeding, angina or cardiac hospitalisation. Indicated events were verified by assessing patients’ medical records. The study was conducted according to the principles of the Declaration of Helsinki and in accordance with the Medical Research Involving Human Subjects Act. A waiver for written informed consent was provided by the local ethics committee.

### Data collection

Information was obtained from patients’ medical records or retrieved from patients’ general practitioner. Collected patients’ characteristics included sex, age, diabetes mellitus, creatinine (mmol/l), history of CABG, history of atrial fibrillation, location of lesions, completeness of revascularisation (determined by location of lesions and revascularised vessels through reviewing the pre-procedural angiogram, procedural angiogram (PCI) and revascularisation reports (PCI and CABG) by at least two qualified researchers), type of stent implanted (bare-metal stent, second generation drug-eluting stent, bioresorbable vascular scaffold (BVS)), urgency of procedure and Euroscore I. Also, the national mortality register was consulted.

### Definitions

A stenosis of ≥70% or fractional flow reserve measurement <0.80 was considered significant in a coronary vessel of ≥2.0 mm in diameter. A left main stenosis was considered significant when ≥50%. Multivessel disease was presence of a significant stenosis in the left main or at least two major coronary arteries. A procedure was considered elective when it was scheduled and performed on patients with stable coronary artery disease, urgent when it was performed in context of an acute coronary syndrome (ACS) and emergency when it was performed immediately because of the acute nature of the medical condition and increased morbidity or mortality associated with temporary delay in treatment [7]. Completeness of revascularisation was determined as treatment of all significant lesions. ACS was defined according to the Fourth Universal Definition of myocardial infarction or unstable angina [8]. CVA was described as acute new neurological deficit by ischaemic stroke which lasted >24 h or ended in death within 24 h, excluding haemorrhagic CVAs. Repeat revascularisation was defined as revascularisation with either PCI or CABG unless index treatment was scheduled as a staged procedure. In the absence of questionnaires, the following outcome measures were chosen to provide an indication of quality of life: recurrent angina, cardiac rehospitalisation and new onset atrial fibrillation. Recurrent angina was classified according to the Canadian Cardiovascular Society of Angina Grading scale. Angina definitely provoked by other causes e.g. anaemia or tachycardia was excluded. Angina was further subdivided into documented ischaemia, which included either positive electrocardiogram exercise testing, stress imaging or when adjustment of pharmaceutical therapy for angina relieved the symptoms. Cardiac rehospitalisation was specified as readmission after the procedure for any cardiac cause, e.g. heart failure or atrial fibrillation. New onset atrial fibrillation was captured when it occurred after the procedure and remained after discharge or presented post-discharge. Bleeding was classified according to Bleeding Academic Research Consortium (BARC) criteria, we captured BARC bleeding type 3 and 5 [9].

### Outcome

Primary outcome was all-cause mortality. We also captured ACS, CVA, recurrent angina, repeat revascularisation, cardiac rehospitalisation, new onset atrial fibrillation and bleeding events.

### Statistical analysis

Baseline characteristics were compared using Student’s t‑test or Mann-Whitney U test for continuous variables and chi-squared test for binary variables. Continuous data were expressed as mean ± standard deviation (SD). Categorical variables were described as frequencies and percentages. Unadjusted primary and secondary outcomes were presented as Kaplan-Meier curves, differences were assessed by using the log-rank test. Risk-adjusted hazard ratios (aHR) with 95% confidence intervals (CI) were estimated by Cox proportional hazard regression. Baseline variables with a *p*-value <0.100 in the univariate analysis were included in the multivariate analysis. *P* < 0.05 was considered statistically significant.

## Results

**Fig. 1:**
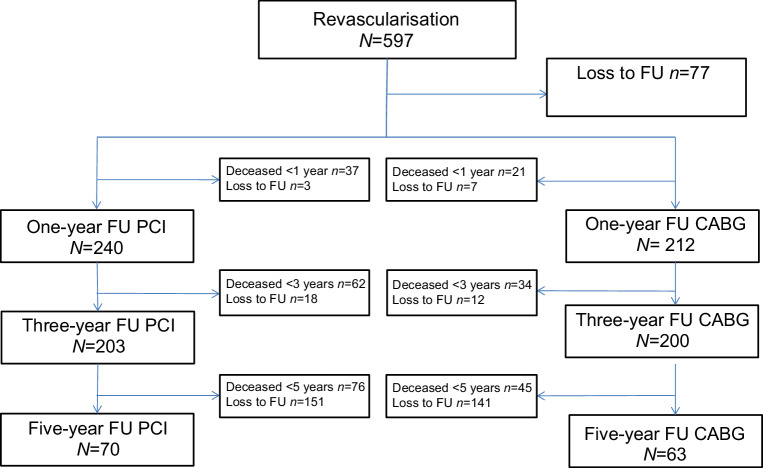
Flowchart follow-up. *CABG* coronary artery bypass grafting,* FU* follow-up, *PCI* percutaneous coronary intervention

We included a total of 597 patients; 346 in the PCI group and 251 in the CABG group. Median follow-up period was 4 years (interquartile range [IQR] 2.8–5.3 years). Baseline characteristics are presented in Tab. 1. Patients who underwent PCI were older; 54% of patients in the PCI group were aged ≥80 vs 39% in the CABG group (*p* < 0.001). Patients in the PCI group more frequently had a previous medical history of CABG (21 vs 5.6%, *p* < 0.01) and more often needed urgent revascularisation (25 vs 17%, *p* = 0.024) compared with patients in the CABG group. Patients who underwent CABG were more often male (73 vs 65%, *p* = 0.041), had more coronary segments involved, and had left main disease more frequently (28 vs 14%, *p* < 0.01) compared with patients who underwent PCI. Complete revascularisation was more frequently achieved in patients undergoing CABG than in patients undergoing PCI (71 vs 30%, *p* < 0.01). Incidence of diabetes mellitus and serum creatinine levels were similar in both groups. Among PCI patients, the majority received a drug-eluting stent (89%). Most CABG patients received a left internal mammary artery graft (94%). Mean Euroscore I in the CABG group was 8.6. Loss to follow-up of the study population is presented in Fig. 1. For the primary outcome we checked the national mortality register. Therefore, only 19 patients were lost to follow-up.

**Table 1:** Baseline characteristics

Characteristics	PCI (*N* = 346)	CABG (*N* = 251)	*P*-value
Male gender—*N* (%)	225 (65)	183 (73)	0.041
Age—year mean ± SD	80 ± 3.9	79 ± 3.4	<0.001
Age ≥80—*N* (%)	186 (54)	98 (39)	<0.001
Diabetes—*N* (%)	93 (27)	75 (30)	0.433
Creatinine µmol/l mean ± SD	109 ± 85	102 ± 42	0.246
Creatinine ≥200 µmol/l—*N* (%)	10 (3.2)	6 (2.4)	0.573
History of CABG—*N* (%)	71 (21)	14 (5.6)	<0.001
History of AF—*N* (%)	43 (12)	23 (9)	0.125
Status elective—*N* (%)	260 (75)	208 (83)	0.024
Status urgent—*N* (%)	86 (25)	43 (17)	
Complete revascularisation—*N* (%)	102 (30)	179 (71)	<0.001
**Coronary artery disease**			
LAD >70%—*N* (%)	263 (76)	233 (93)	<0.001
RCx >70%—*N* (%)	231 (67)	192 (77)	0.010
RCA >70%—*N* (%)	236 (68)	192 (77)	0.027
LM >50%—*N* (%)	48 (14)	70 (28)	<0.001
Single LM disease—*N* (%)	12 (3.5)	3 (1.2)	0.080
LM + 1VD—*N* (%)	20 (5.8)	14 (5.6)	0.916
LM + 2VD—*N* (%)	4 (1.2)	30 (12)	<0.001
LM + 3VD—*N* (%)	12 (3.5)	23 (9.2)	0.003
2VD—*N* (%)	228 (66)	69 (28)	<0.001
3VD—*N* (%)	70 (20)	112 (45)	<0.001
**PCI characteristics**			
DES—*N* (%)	309 (89)		
BMS—*N* (%)	27 (7.8)		
BVS—*N *(%)	2 (0.6)		
Balloon—*N* (%)	81 (23)		
Number of stents mean ± SD	1.71 ± 1.0		
**CABG characteristics**			
Euroscore I mean ± SD		8.6 (7.7)	
LIMA—*N* (%)		235 (94)	

*AF* atrial fibrillation, *BMS* bare-metal stent, *BVS* bioresorbable vascular scaffold, *CABG* coronary artery bypass grafting; *DES* drug-eluting stent, *LAD* left anterior descending artery, *LM* left main, *PCI* percutaneous coronary intervention, *RCA* right coronary artery, *RCx* ramus circumflex artery, *SD* standard deviation, *VD* vessel disease

### Mortality

**Fig. 2:**
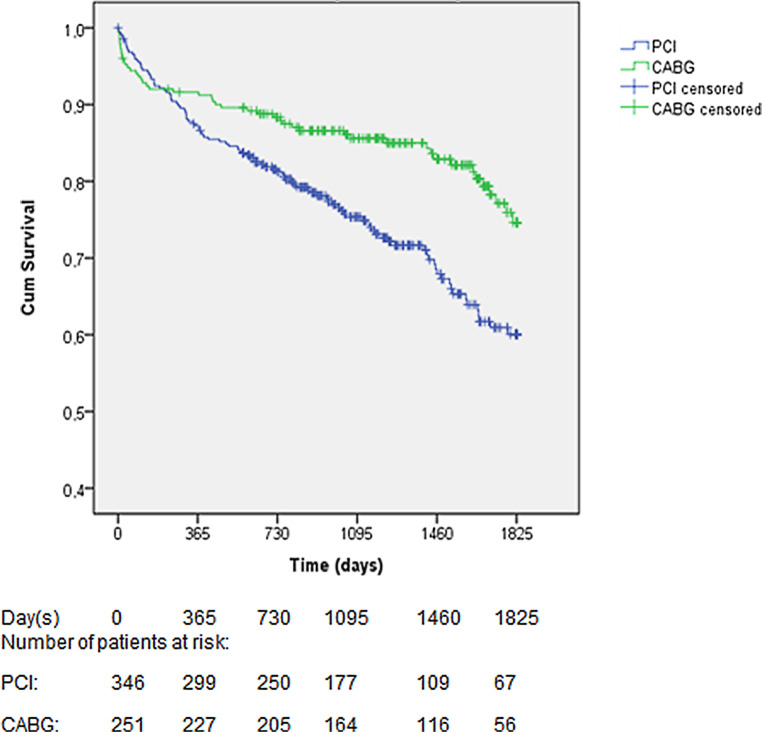
Kaplan-Meier survival curve for primary outcome. *CABG* coronary artery bypass grafting, *PCI* percutaneous coronary intervention

The unadjusted analyses showed a significantly higher long-term mortality rate after PCI than after CABG (39.9% vs 25.4%, *p* = 0.001; Fig. 2). Cox-regression analysis revealed older age, higher creatinine and left main disease to be independent predictors of long-term mortality. After adjustment for these predictors, 5‑year mortality remained significantly higher after PCI (aHR 1.59 [95% CI 1.10–2.28], *p* = 0.013).

### Individual outcomes

**Fig. 3:**
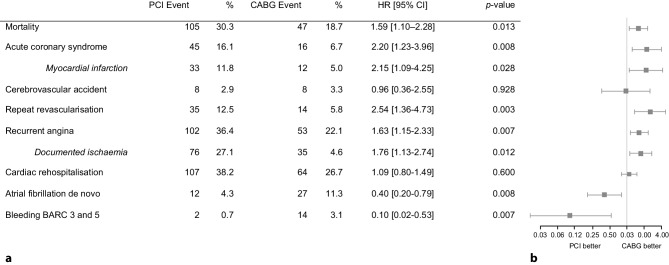
Five-year outcomes after PCI and CABG. *BARC* Bleeding Academic Research Consortium, *CABG* coronary artery bypass graft, *CI* confidence interval, *HR* hazard ratio, *PCI* percutaneous coronary intervention

After adjustment for the concerning independent predictors, recurrent ACS, consisting of myocardial infarction in 73% of cases (aHR 2.20 [95% CI 1.23–3.96], *p* = 0.008), repeat revascularisation (aHR 2.54 [95% CI 1.36–4.73], *p* = 0.003) and recurrent angina (aHR 1.63 [95% CI 1.15–2.33], *p* = 0.007), all occurred more frequently in patients who underwent PCI compared with CABG. On the other hand, new onset atrial fibrillation (aHR 0.40 [95% CI 0.20–0.79], *p* = 0.008) and bleeding (aHR 0.10 [95% CI 0.02–0.53], *p* = 0.007) occurred significantly more often in patients who underwent CABG. The incidence of CVA and cardiac rehospitalisation was comparable between both groups (Fig. 3).

### Recurrent angina

**Fig. 4:**
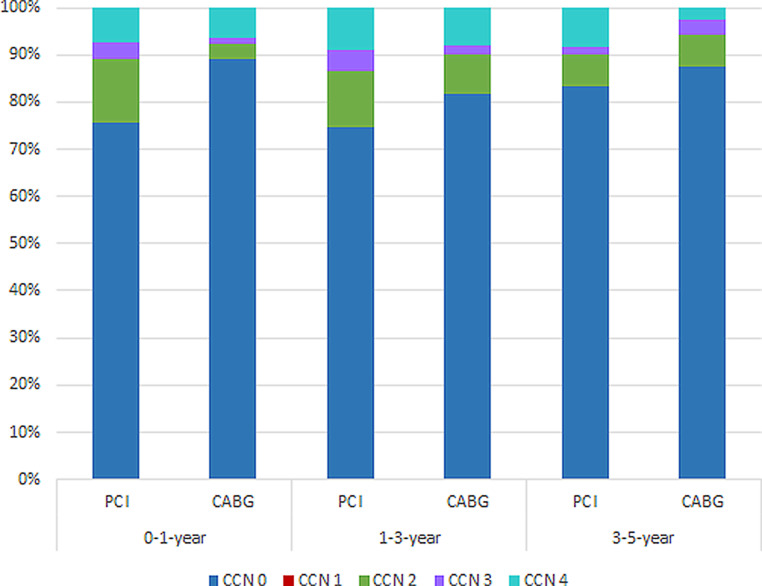
Recurrent angina classified according to the Canadian Cardiovascular Society of Angina grading scale: Grade 0 asymptomatic/absent angina; Grade I angina only with strenuous exertion; Grade II angina with moderate exertion; Grade III angina with mild exertion; Grade IV angina at rest. *CCN* Cardiac Care Network of Ontario

Recurrent angina during first year after index procedure developed more often in PCI patients compared with CABG patients (24.4 vs 9.2%, *p* < 0.001). The difference between the two groups, however, decreased during follow-up (Fig. 4). Recurrent angina was caused by documented ischaemia in 72% of cases, and differed significantly in favour of CABG (37.9 vs 20.2%, *p* < 0.012).

## Discussion

In this large and unselected registry of patients aged 75 years or older with multivessel disease or left main disease treated with PCI or CABG, we identified higher mortality after PCI than after CABG. In addition, we found patients undergoing PCI to have higher risk of ACS, recurrent angina, and repeat revascularisation during follow-up. We expected to find completeness of revascularisation to be an independent predictor of adverse outcomes. However, in this analysis we could not confirm this hypothesis.

Studies with long-term follow-up of PCI versus CABG in elderly patients are scarce, and outcomes are inconsistent. In our study, mortality appeared to be higher after PCI than after CABG. This is in accordance with results from Nicolini et al. who compared PCI with CABG and included 1388 patients of ≥80 years with multivessel disease and/or left main disease. They also found better survival after CABG than after PCI, although this was not statistically significant [6]. On the other hand, Sheridan et al. who included very old patients of ≥85 years with multivessel disease and presentation with ACS, found a significant benefit of CABG compared with PCI already after 2 years [10]. These differences in outcome between the two studies could have been caused by differences in baseline characteristics; the patients Nicolini included were younger than the ones included in Sheridan’s study and were predominantly treated with a bare-metal stent, while drug-eluting stents were used in the study of Sheridan.

It is, however, debatable whether elderly patients value survival as the most important goal of revascularisation. Therefore, we assessed recurrent angina, cardiac rehospitalisation and repeat revascularisation after CABG and after PCI. These outcomes occurred significantly less frequent as early as one year following CABG as compared with PCI. This is consistent with the literature where target vessel revascularisation and heart failure hospitalisations occurred significantly less frequently in the CABG group compared with the PCI group [6, 11]. This difference in repeat revascularisation and hospitalisation could be explained by more frequently occurring failure of revascularisation (restenosis) after PCI than after CABG (graft failure) or by more complete revascularisation after CABG than after PCI. However, the latter is not corroborated by our study, where incomplete revascularisation was not an independent predictor of death or major adverse cardiac and cerebral events.

The 2018 ESC guideline on myocardial revascularisation recommends prioritising completeness of revascularisation when deciding between CABG and PCI, based on a meta-analysis of 35 randomised controlled trials and observational studies [12]. Complete revascularisation was associated with reduced long-term mortality compared with incomplete revascularisation which was observed both after CABG and after PCI. However, evidence concerning revascularisation in octogenarians showed conflicting results [13, 14]. In these elderly patients, it is suggested that complete revascularisation is not necessary to provide good long-term prognosis. This is supported by Généreux et al. who, based on SYNTAX score, identified 70% completeness of revascularisation to be sufficient to provide comparable long-term prognosis to 100% completeness of revascularisation [15].

A strength of this study is the consistency and uniformity of both procedures during the study period in our centre, e.g. same decision making process in the heart team, similar and contemporary revascularisation methods (performed by the same surgeons and cardiologists), and the medical treatment after both revascularisation methods was according to the same hospital protocols. This suggests that effects found in this study, are truly attributable to the revascularisation method, while this may be different in multicentre studies. In addition, all coronary angiograms were reviewed and compared with revascularisation reports to ascertain completeness of revascularisation.

Some important limitations of this study should also be discussed. First, the retrospective design of the study may have resulted in selection bias allocating patients to one of the two revascularisation strategies. By using adjustment through Cox-regression we tried to correct for the differences in baseline variables. However, we should take into account that this still could have had an influence on the results. In addition, in both groups we may have included patients who had an absolute contraindication for the other revascularisation strategy. Second, we were unable to measure quality of life. Quality of life is an important measure, especially at advanced age, and could differ between PCI and CABG patients, as the recovery and rehabilitation period after CABG is longer and more intense than after PCI. However, we evaluated recurrent angina and rehospitalisation as substitute outcomes, capturing, in our view, important aspects of quality of life.

To conclude, in this observational study, long-term mortality was higher in elderly patients of 75 years or older with multivessel disease undergoing PCI as compared with CABG. In addition, patients undergoing PCI had a higher risk of ACS, repeat revascularisation and recurrent angina.
